# Prevalence, Screening, and Parental Awareness of Oral Human Papillomavirus in Pediatric Populations (HOPE Project): Findings from a Cross-Sectional Pilot Study

**DOI:** 10.3390/jcm14082808

**Published:** 2025-04-18

**Authors:** Vera Panzarella, Giuseppina Campisi, Giuseppina Capra, Arianna Sucato, Viviana D’Arpa, Giuliana Minacapilli, Gaetano La Mantia, Laura Maniscalco, Monica Bazzano, Elena Consiglio, Giovanna Giuliana

**Affiliations:** 1 Department of Precision Medicine in Medical, Surgical and Critical Care (MePreCC), University of Palermo, 90127 Palermo, Italy; vera.panzarella@unipa.it (V.P.); viviana.darpa@you.unipa.it (V.D.); giuliana.minacapilli@you.unipa.it (G.M.); monica.bazzano@unipa.it (M.B.); giovanna.giuliana@unipa.it (G.G.); 2 Center for Sustainability and Ecological Transition (CSTE), University of Palermo, 90127 Palermo, Italy; 3 Department of Biomedicine, Neuroscience and Advanced Diagnostics (BiND), University of Palermo, 90127 Palermo, Italy; giuseppina.campisi@unipa.it; 4 Unit of Oral Medicine and Dentistry for Fragile Patients, Department of Rehabilitation, Fragility, and Continuity of Care, University Hospital Palermo, 90127 Palermo, Italy; 5 Department of Health Promotion, Mother and Child Care, Internal Medicine, and Medical Specialties (ProMISE), University of Palermo, 90127 Palermo, Italy; giuseppina.capra@unipa.it (G.C.); arianna.sucato@unipa.it (A.S.); laura.maniscalco04@unipa.it (L.M.); 6 Department of Biomedical and Dental Sciences and Morphofunctional Imaging, University of Messina, 98122 Messina, Italy; 7 Department of Law (Di.Gi), University of Palermo, 90134 Palermo, Italy; elena.consiglio@unipa.it

**Keywords:** health knowledge, human papillomavirus, human papillomavirus DNA test, papillomavirus detection, pediatrics, social determinants of health

## Abstract

**Background**: Human papillomavirus (HPV) infection in pediatric populations is an emerging area of interest due to its potential role in early viral transmission and long-term oncogenic risk. The oral cavity may serve as a reservoir for high-risk HPV types; however, its prevalence in children remains under-investigated and unclear. The HOPE (**H**uman papillomavirus **O**ral infection in **PE**diatric population) project aims to assess the prevalence of oral HPV using an innovative salivary sampling method, also exploring the influence of social determinants on parental awareness and attitudes toward HPV vaccination. This paper presents the findings from a cross-sectional pilot study. **Methods**: This pilot investigation included a total of 70 children (0–14 years) who underwent an oral examination and non-invasive salivary sampling using the novel LolliSponge device. HPV detection was performed using INNO-LiPA^®^ HPV genotyping. Parents completed a questionnaire assessing socio-demographic factors and HPV-related knowledge. Associations between oral health status, social determinants, and HPV awareness were analyzed. **Results**: The LolliSponge device demonstrated excellent acceptability among the pilot population. The mean age at recruitment of the children thus far (8 years) appears to correlate with the absence of oral HPV infection. Regarding parental knowledge and attitudes, 51 out of the 70 respondents (72.9%, 95% CI: 62–83%) reported having heard of HPV; however, 94.3% (66/70) were unaware of its transmission routes, and 60% (42/70) did not know that it can cause cancer. Only 4 out of the 70 participants (5.7%) reported that their child had received the HPV vaccine. Lower awareness of the HPV vaccine was significantly associated with a lower educational level (*p* = 0.001), being married (*p* = 0.03), and having three or more children (*p* = 0.039). Awareness of the vaccine’s existence also varied significantly by parental occupation (*p* = 0.02). **Conclusions**: The pilot findings of the HOPE project highlight both the potential of innovative strategies for detecting oral HPV infection in children and critical gaps in parental knowledge and vaccine uptake. Preliminary data also reveal an age-related bias in HPV status, suggesting the need for further investigations in a larger cohort of younger children (<5 years).

## 1. Introduction

Human papillomavirus (HPV) is a group of viruses comprising more than 200 identified types, several of which are linked to various cancers, including cervical, anal, and oropharyngeal cancers. Traditionally, HPV has been associated with genital infections transmitted through sexual contact [1]. However, recent studies have highlighted its presence in the healthy oral cavity, raising questions about its transmission, clinical significance, and public health implications [2,3,4]. The prevalence of oral HPV infection in children is less investigated than in adults, varying based on demographic and socio-economic factors and, most importantly, on HPV detection procedures [5,6,7,8,9].

After oral viral exposure, while most pediatric HPV infections are transient and resolve spontaneously, with or without clinical benign manifestations (e.g., oral warts, papillomas, and condylomas), a subset of children may develop persistent infections [10]. This condition could have a potential impact on future cancer risk. Several factors contribute to the persistence of HPV infection in children, including viral factors such as HPV genotype (high-risk (HR) HPV vs. low-risk (LR) HPV), host factors such as immune status and genetic predisposition, and environmental factors such as exposure to tobacco smoke or other carcinogens [11,12,13]. In a study by Mammas et al. in 2013, the term “Trojan horse oncogenic strategy” was first used to describe the concept of children representing a reservoir of HR-HPV infection, similar to the Trojan horse in Greek mythology [14].

Most persistent silent mucosal HR-HPV infections in infants are oral or oropharyngeal, and these conditions may represent the initial step in the development of multifocal cancer [12,14,15]. These processes are strongly influenced by poor oral health, which contributes to infectious–inflammatory diseases such as gingivitis [16]. This, in turn, may partly explain the high prevalence of rare HPV-related oral carcinomas, particularly in individuals of developmental age [16,17]. For these reasons, the prevalence and clinical significance of oral HPV infection in children are areas of growing interest and concern.

Diagnosing HPV infection in children presents several challenges, including the lack of standardized sampling methods, difficulty in obtaining appropriate samples, and the potential for false positive results due to contamination or cross-reactivity with non-oncogenic HR-HPV types [18].

Currently, no FDA-approved tests are available for detecting HPV DNA or RNA in saliva. However, salivary rinse samples are considered the “gold standard” in research settings for assessing oral HPV infection in both cancer patients and healthy individuals [18,19]. Despite their efficacy, these samples are not commonly used in children due to poor tolerance, as well as an increased risk of contamination and concerns regarding sample appropriateness, which are more significant compared to adults.

Additionally, the complete transmission pathways of oral HPV in children have not been fully elucidated and may include vertical transmission (mother-to-child), non-sexual contact, orogenital contact, and autoinoculation [20,21,22,23]. Therefore, evaluating the child’s family context is crucial for understanding their exposure to HPV infection risks and for appropriate monitoring.

Moreover, despite the availability of preventive strategies such as HPV vaccination and educational programs on infection risks, significant socio-cultural barriers hinder their widespread adoption [24,25,26].

Specific “*social determinants of health*”, particularly socio-economic status, religion, education level, age, and gender, contribute to defining the environment in which a person lives. This environment is also shaped by the ability of an individual to mitigate health risks, for example, by having the knowledge and economic resources to relocate to a healthier environment or by accessing the appropriate preventive healthcare, such as HPV vaccination [27,28].

In this scenario, understanding the factors contributing to the status and persistence of HPV infection, improving detection and diagnostic methods, and implementing preventive strategies are essential steps in mitigating the burden of HPV-related diseases in children.

Considering these challenges, we contextualize our project: “**H**uman Papillomavirus **O**ral infection in **PE**diatric population” (HOPE). HOPE primarily aims to be a large cross-sectional study of the pediatric population, designed to better understand the rate and determinants of postnatal HPV infection and transmission, as well as the rate and determinants of viral status and persistence, particularly in relation to oral health. The secondary aim is to highlight the impact of “social determinants of health” and the level of awareness/knowledge about HPV infection on access to currently available preventive strategies, including HPV vaccination. 

To the best of our knowledge, this is the first time that the clinical, instrumental, and epidemiological/socio-cultural variables related to oral HPV status in the pediatric population have been evaluated together. The project will initially focus on the pediatric population of Palermo, a city representative of the socio-cultural context of Southern Italy, and will later extend the findings to other areas, taking local specificities into account.

This paper presents the findings from the cross-sectional pilot phase of the HOPE project, which evaluates the feasibility and acceptability of an innovative salivary sampling technique for detecting oral HPV in pediatric populations. Additionally, it examines the socio-economic and cultural context of families, assessing their awareness, knowledge, and perceptions of HPV infection.

## 2. Materials and Methods

### 2.1. Study Design and Participants

The HOPE project received approval from the Clinical Research Ethics Committee (report no. 4 dated 12 January 2024). The cross-sectional study consisted of children who attended our Dentistry Unit (University Hospital Palermo) for dental treatments from January to July 2024, and their mother or father. Written informed consent was obtained from all the parents participating in this study.

### 2.2. Inclusion and Exclusion Criteria

The inclusion criteria included the following:

- Age between 0 and 14 years.
- Absence of mental or cognitive disabilities.
- No history of previous dental treatments.
- No clinical need for urgent dental care at the time of enrolment.
- Ability to cooperate with the procedures required for oral examination and saliva sampling.

The exclusion criteria included the following:

- Presence of mental or physical disabilities that could interfere with cooperation.
- Uncooperative behavior (refusal to perform saliva collection using the LolliSponge device).
- The presence of congenital oro-facial anomalies, such as cleft lip and/or palate, which could compromise the correct use of a sponge device.

### 2.3. Oral Health Assessment

The oral status was recorded from all the recruited children. Particularly, gingiva and teeth were examined for gingivitis and caries according to Word Health Organization (WHO) criteria. Gingivitis was classified into absent, localized (defined as a Bleeding on Probing [BoP] score between 10% and 30% of present and probeable teeth), generalized (defined as Bleeding on Probing [BoP] score > 30% of present and probeable teeth), and associated with orthodontic treatment. Early Childhood Caries (ECC) was determined on deciduous dentition, distinguishing for types: absent, ECC type I (mild/moderate: isolated carious lesions on incisors and/or molars), ECC Type II (moderate/severe: involving carious lesions on the approximal surfaces of the upper incisors, with or without involvement of the molars), and ECC Type III (severe: carious lesions affecting all the dental elements, including mandibular incisors). Caries in mixed or permanent dentitions were recorded using the International Caries Detection and Assessment System (ICDAS) classification and distinguishing four categories: absent (ICDAS 0), initial (ICDAS 1–2), moderate (ICDAS 3–4), and extensive (ICDAS 5–6). The oral examination was performed by postgraduate dental trainees specializing in pediatric dentistry (V.D. e G.M).

Following the oral examination, children underwent non-invasive saliva sampling for oral HPV detection using the LolliSponge™ device (Copan Italia S.p.A., Brescia, Italy). Originally developed for self-salivary sampling in COVID-19 surveillance programs, the device has been validated for its diagnostic accuracy in HPV detection when compared with the “gold standard” salivary sample (oral rinse) in a highly representative patient population for oral HPV infection (oral oncology patients) [29].

Given the strong concordance observed between the two techniques, this salivary device was selected to ensure both the compliance and adequacy of oral HPV sampling in this specific pediatric setting [29].

To enhance compliance among children and to ensure correct sampling with the LolliSponge™ device, saliva was collected following the viewing of a dedicated instructional video developed by the manufacturer specifically for the pediatric population (https://www.youtube.com/watch?v=XXw3UTgagFM; accessed on 8 April 2025).

**Figure 1:**
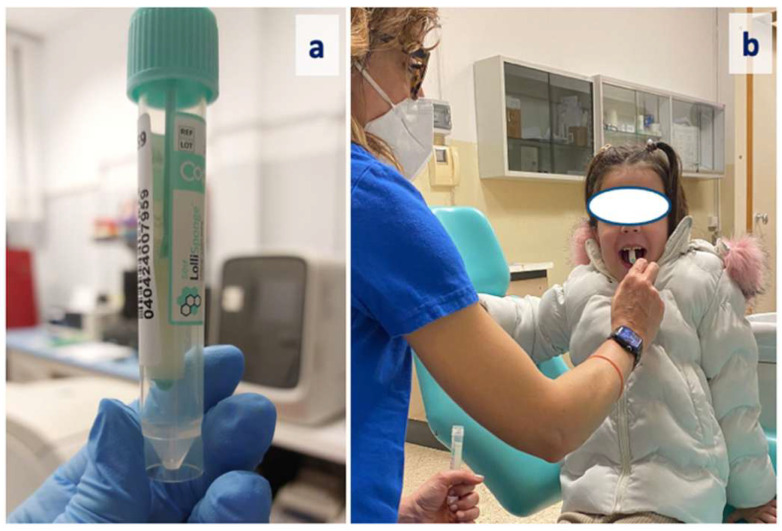
LolliSponge^TM^ sponge device (**a**) and salivary sampling procedure (**b**).

Behind insertion into the mouth, the sponge device was gently moved on the tongue and between the teeth and cheeks for 1 min (Figure 1).

### 2.4. Oral Sample Processing, DNA Extraction, and HPV DNA Detection

Each sponge was sent to the Microbiology and Virology Unit for microbiological analysis. The samples were stored at 2–8 °C and processed either immediately or within 24 h. Upon receipt, the sponges were immediately centrifuged at 450× *g* for 60 s to extract saliva in accordance with the manufacturer’s instructions (Copan Italia S.p.A., Brescia, Italy).

Each sample was then subjected to the DNA extraction process, performed using the magnetic bead technology of the ELITe InGenius^®^ automatic extractor (Elitechgroup, Turin, Italy). The automated nucleic acid extraction and purification were carried out using the reagent ELITe InGenius SP 200 following the manufacturer’s instructions. The reagent set isolated nucleic acids from 200 µL samples using magnetic beads for nucleic acid capture.

HPV-DNA detection was performed on each eluate using the INNO-LiPA^®^ HPV Genotyping Extra II kit (Fujirebio, Tokyo, Japan), which employs PCR amplification with biotinylated primers followed by a reverse dot blot hybridization assay. The manufacturer’s instructions were followed throughout the procedure.

The INNO-LiPA^®^ HPV Genotyping Extra II technique enables the identification of 32 HPV genotypes: 12 low-risk HPV genotypes (HPV6, HPV11, HPV40, HPV42, HPV43, HPV44, HPV54, HPV61, HPV62, HPV81, HPV83, and HPV89) and 20 high-risk HPV genotypes (HPV16, HPV18, HPV31, HPV33, HPV35, HPV39, HPV45, HPV51, HPV52, HPV56, HPV58, HPV59, HPV67, HPV68, HPV26, HPV53, HPV66, HPV70, HPV73, and HPV82).

The PCR reaction simultaneously amplifies two targets: a 65 bp region of the HPV L1 gene using SPF10-based primers, and HLA-DBP1 as an endogenous control for sample adequacy. In the reverse dot blot hybridization step, the PCR products were bound to DNA probes immobilized on a membrane strip. The biotin–streptavidin system allows us to reveal the endogenous control probe, the genotype-specific probe, and two additional generic “HPV control” probes.

Each sample was subjected to a nested PCR based on a first amplification step with the PGMY09/11 primer pair followed by a second step with the GP05+/GP06+ primers. This procedure allows the detection of over 200 HPV genotypes, including those not covered by the list of 32 HPV genotypes in the INNO-LiPA^®^ HPV Genotyping Extra II. Through this process, we assessed the true negative nature of the samples, as described elsewhere [30].

The entire oral HPV sampling and detecting protocols by the LolliSpong^TM^ device, along with the diagnostic accuracy results, are described in our previous study, which has been recently published [29].

### 2.5. Questionnaire

The questionnaire used in this study was developed ad hoc for the pilot phase of the HOPE project. Its aim was to assess the feasibility, clarity, and acceptability of the items in the context of a broader epidemiological investigation. Minor revisions were made based on participant feedback during its preliminary administration, comprising cultural and linguistic adaptations of the questionnaire for the Palermo population. Formal validation of the instrument is planned for subsequent project phases in a definitive participant sample.

All relatives (mother or father) of the children who were subjected to the examination and oral sampling were invited to complete a specific questionnaire, divided into two sections. The socio-demographic data were collected in the first section, comprised 9 items: age (scored into 18–25; 26–33; 34–41; 42–49; and >50), nationality (Italian; other), education level (high or low) employment (homemaker; unemployed; freelancer; employed; or pensioner), parental status (single; cohabiting; separated/divorced; widowed; or married), religion (Catholic; other), income (unknown; low (up to EUR 36,151.98); medium (EUR 36,151.99–EUR 70,000); or high (EUR 70,000.01–EUR 100,000), residence (small city: less than 100,000 inhabitants; medium city: 100,000 to 500,000 inhabitants; or large city: over 500,000 inhabitants), and number of children (1–2; ≥3).

The second section contained 15 specific questions regarding parents’ knowledge of HPV infection, particularly its transmission, and their attitudes toward its prevention, listed below (with possible answers):

- (for females only) Do you undergo gynecological examinations periodically? (Yes, at least once a year; Yes, at least once every 3 years; Yes, rarely, and only if necessary; No, only during childbirth/births; Not applicable, I am male).
- (for females only) Have you ever had a PAP test? (Yes, at least once every 3 years; No, but I recognize its importance for the prevention of cervical cancer; No, and I don’t think I need to; No, I am not aware; Not applicable, I am male).
- Do you regularly visit the dentist? (Never; Yes, at least once a year; Yes, at least once every 3 years; Yes, rarely, and only if necessary).
- Have you ever heard of the Human Papillomavirus (HPV)? (No; Yes).
- If YES, what are the transmission methods? Multiple choices are allowed [I am not aware; Between partners, through direct sexual contact (from the infected part—vagina, penis, anus, mouth—to the other); Between partners, indirectly sexually (through semen, blood or infected saliva); From mother to child across the placenta; From mother to child through childbirth; From mother to child through breastfeeding; From one person to another, through infected skin; From one person to another, through the sharing of infected hygiene devices or dishes].
- Which of the following sexual habits/practices increase the risk of contracting HPV, in the various areas (ano-genital and oral)? Multiple choices are allowed [I am not aware; Deep kisses; Large number of sexual partners; Unprotected sex practices (inconsistent use of condoms and/or oral dam)].
- Which of the following non-sexual habits/practices increase the risk of contracting HPV? Multiple choices are allowed [I am not aware; Sharing of linen and clothing that have encounter infected people; Blood transfusions/sharing infected syringes; Voluptuary habits (tobacco, alcohol); Childbirth;].
- Do you know that HPV can cause cancer? (Yes; No). If YES, which of these? *Multiple choices are allowed* (I am not aware; cancer; Yes, cervical cancer; Yes, vulvar and vaginal cancer; Yes, penile cancer; Yes, anal cancer; Yes, oropharyngeal cancer; Yes, but other tumors than those listed).
- HPV infection can be contracted from (I am not aware; Men; Women; Both;).
- Is there a medical therapy to defeat HPV infection? (I am not aware; No; Yes;).
- Are you aware of the HPV vaccine? (No; Yes).
- If YES, who should it be administered to? *Multiple choices are allowed* [To everyone (M, F) between 11 and 12 years old; To everyone (M, F), at any age; Only to females, between 11 and 12 years old; Only to females, at any age].
- Are you vaccinated against HPV? (No, I am not aware that there is a vaccine; No, I’m not interested; No, I am not vaccinated; I couldn’t say if I’m vaccinated; Yes; No, because it is not mandatory; No, I am afraid of the HPV vaccination; No, I am against HPV vaccination; No, I’m afraid of vaccinations in general; No, I am against vaccinations in general; No, after the anti-COVID vaccination, I do not want to undergo another type of vaccination;
- If YES, at what age did you complete the vaccination? (By age 25; After age 25).
- Will you have your child vaccinated against HPV? [No, I don’t think it’s useful; No, because it is not mandatory; No, I am afraid of HPV vaccination for him/her; No, I am against HPV vaccination for him/her; No, I’m afraid of vaccinations in general for him/her; No, I am against vaccinations in general for him/her; No, after the anti-COVID vaccination, I do not want to subject my children to any other type of vaccination; Now I do not have adequate information to decide (I’ll do when the physicians tell me); Yes, I will have him vaccinated between 11 and 12 years old (as foreseen in the optional vaccination plan); Yes, I will get him/her vaccinated when he/she asks me].

### 2.6. Statistical Analysis

A descriptive analysis was conducted, along with an aggregation of responses, to highlight any statistically significant associations. Categorical variables were presented as counts and percentages. To assess whether the child’s oral health status, attitudes towards care and health, and the parents’ knowledge regarding HPV infection and preventive measures were associated with specific “social determinants of health”—such as education level, socio-economic status, marital status, place of residence, type of housing, occupation, and number of children—either the chi-square test or Fisher’s exact test was applied depending on suitability. Specifically, Fisher’s exact test was used instead of the chi-square test when the expected frequency of one or more cells was less than 5.

All the tests with a two-sided *p*-value below 0.05 were considered statistically significant. The entire statistical analysis was performed using the R software (version 4.3.3) to ensure a rigorous examination of the data and support the identification of meaningful patterns and associations [31].

## 3. Results

Seventy pediatric patients were recruited (average age 8 years). A high level of participant adherence was observed (90%), defined as the proportion of informed families about the HOPE project who agreed to participate in the study and completed all the planned procedures (oral examination, saliva collection, and questionnaire). In Table 1 we reported the oral status of children’s samples in the pilot study.

The HPV sampling using LolliSponge^TM^ was well accepted and enjoyed by all the enrolled children (the compliance rate was 100%), particularly after watching the dedicated instructional video.

The pilot screening questionnaire mainly involved accompanying mothers (62 [88.6%]), housewives (48 [68.6%]), aged between 34 and 41 years (30 [42.9%]). Some categorical variables were combined to avoid sparse data. Religion was categorized into “Catholic”, which includes practicing and non-practicing Catholics, and “Other”, which includes Muslims, atheists/agnostics, and practitioners of other religions. The variable education included initially the categories “primary school diploma”, “middle school diploma”, “high school diploma”, “university degree” and “post-degree”. This variable was then dichotomized into “High”, which includes those with a high school diploma, university degree, or post-graduate degree, and “Low”, which includes those with a primary or middle school diploma.

**Figure 2:**
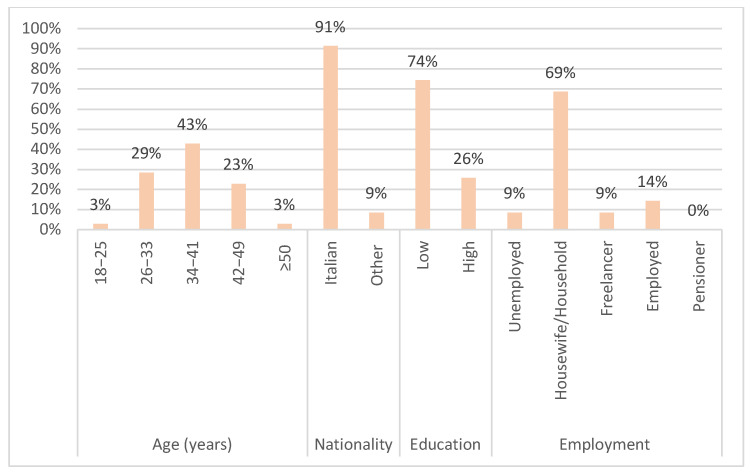
Demographic and employment characteristics of pilot population.

**Figure 3:**
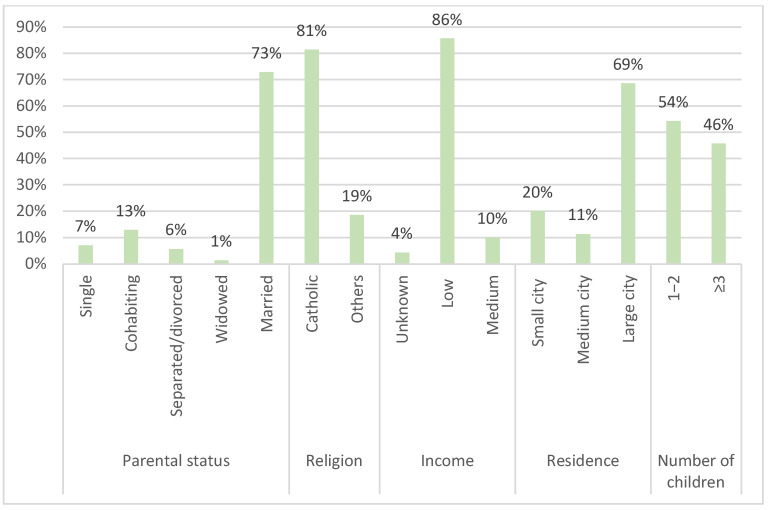
Parental status, religion, income, residence, and family size of pilot population.

Most of the respondents showed a low average level of education (52 of 70 [74.3%]) and reported a low average socio-economic status. A total of 57 out of the 70 respondents (81.4%) were Catholic (either practicing or not), 60 out of the 70 (85.8%) were married or cohabiting, and 48 out of the 70 (68.6%) lived in a large city (Figure 2 and Figure 3).

Regarding attitudes toward care, 43.5% of the women interviewed undergo gynecological examinations at least once a year, while 48.4% do so only when necessary or during childbirth. Concerning knowledge of HPV infection and preventive measures, 72.9% [95%CI: 62–83%] of the sample reported having heard of HPV infection. However, most respondents were unaware of its mode of transmission and the habits/practices—both sexual and non-sexual—that promote its spread. Additionally, 60% [95%CI: 49–71%] of the sample were unaware of the link between HPV and cancers, though a significant proportion recognized it as the main risk factor for cervical cancer.

Only 5.7% of the respondents had received the HPV vaccination, but 42.9% stated they would vaccinate their children according to the current guidelines. It is noteworthy that for a considerable number of the respondents, decisions regarding vaccination for their children were contingent upon acquiring more information, particularly from their physicians (Table 2).

To date, since no participants have tested positive for HPV, it is not yet possible to perform statistical analyses on the relationship between oral HPV infection status and the observed dental–periodontal health conditions. However, these conditions were examined in relation to the “social determinants of health” within the family context.

The presence of gingivitis was found to be significantly associated with the type of dentition. Children with mixed dentition had a higher likelihood of presenting gingivitis compared to those with deciduous dentition (OR: 4.38, 95% CI: 1.35–15.40, and *p* = 0.013), as did those with permanent dentition (OR: 4.82, 95% CI: 1.03–29.44, and *p* = 0.046). Regarding parental occupation, gingivitis was more frequently observed among the children of homemakers, with statistically significant differences when compared to the children of unemployed (OR: 0.14, 95% CI: 0.01–0.88, and *p* = 0.036), employed (OR: 0.18, 95% CI: 0.03–0.78, and *p* = 0.022), and self-employed parents (OR: 0.14, 95% CI: 0.01–0.88, and *p* = 0.036), suggesting a possible influence of caregiver status on children’s oral health. (Table 3). Additionally, parental attitudes of scarcity toward oral healthcare were significantly associated with the age group of 42 years and older (OR = 0.19, 95% CI: 0.02–0.95, and *p* = 0.043) (Table 4).

No statistically significant associations were identified between any social determinants of health and the mother’s attitude toward gynecological health or regular Pap test screening. Similarly, no significant associations were found concerning parents’ awareness of HPV infection risks, treatment options, or preventive measures.

Awareness of the HPV vaccine was moderately associated with parental occupation (*p* = 0.02), with homemakers being the most represented group among those aware and unaware of the vaccine. Although not statistically significant, unemployed and self-employed parents were less likely to be aware of the vaccine compared to homemakers (OR = 0.16, 95% CI: 0.01–1.16 for both), whereas employed parents showed higher odds of awareness (OR = 2.68, 95% CI: 0.57–21.16) (Table 5).

Furthermore, the absence of HPV vaccination among relatives was significantly associated with a lower level of education (OR = 6.28, 95% CI: 1.90–25.64; *p* = 0.001), indicating that mothers with lower educational attainment were considerably more likely to report no vaccination (Table 6).

## 4. Discussion

The primary objectives of this pilot study were to define the initial procedural phase of the HOPE (**H**uman Papillomavirus **O**ral infection in **PE**diatric population) project. Specifically, this stage aimed to assess the feasibility of salivary sampling in children, evaluate the acceptability of the LolliSponge^TM^ device, and explore parental knowledge alongside key social determinants relevant to HPV prevention.

### 4.1. Challenges in Oral Pediatric HPV Sampling

Oral rinse and swab methods are traditionally employed for the detection of oral HPV infection, particularly in adolescent and adult populations. However, their application in pediatric settings presents several challenges [32]. Oral rinse requires active cooperation, including the ability to gargle and spit, which is often poorly tolerated or unfeasible among younger children. Swab collection, although less demanding, may cause discomfort or anxiety, and its accuracy is limited by operator variability and difficulties in reaching specific oral sites [33]. Moreover, both methods carry a higher risk of sample contamination, especially in the presence of common pediatric oropharyngeal infections. These limitations highlight the need for alternative, child-friendly sampling tools.

The Copan LolliSponge^TM^ is a novel specimen collection system designed for saliva sampling, particularly useful for diagnostic purposes such as HPV detection. Preliminary results from oral HPV testing showed that the LolliSponge^TM^ has a specificity of 100%, sensitivity of 85.7%, and accuracy of 96.2% compared to oral rinse sampling [29]. These diagnostic performance metrics are promising, especially considering the challenges associated with salivary sampling in young children.

The device simplifies the process by allowing individuals to collect their own saliva samples using a sponge on a stick, which is held in the mouth for a few minutes. This method minimizes contamination risks and does not require rinse/gargle or spitting, making it suitable for self or supported collection, without specific professional assistance [29]. These characteristics are extremely advantageous for a pediatric population, which is notoriously less compliant (especially regarding the oral rinse administration) and susceptible to frequent infections of the respiratory and/or pharyngeal tract. In such cases, the oral rinse may not guarantee contamination-free sample collection [29]. Moreover, it has proven to be a highly effective tool in terms of ease of use and handling. All the children recruited, especially after watching the dedicated video, participated in the sampling process without displaying any discomfort or distress. They even assisted the operator during the sponge suction maneuvers, all of them being pleasantly intrigued by the procedure.

About the oral HPV testing results, the 70 children enrolled to date all tested negative. This finding could be related to the average age of the children recruited so far (8 years), which may be characterized by an immune maturity potentially capable of counteracting the infection or leading to a negative status in individuals following early age [9]. Additionally, the recruitment was limited to healthy children attending a clinical-university setting, potentially representing a low-risk population.

However, only very few studies are available in the literature that explore the prevalence of oral HPV infection in healthy children and adolescents without recruitment filters based on maternal HPV positivity [34,35,36]. These studies report an HPV prevalence range between 0% and 20%, with higher percentages observed in populations under 7 years of age, peaking in the preschool years (19.6%, average age—3 years and 10 months) and using site-specific sampling techniques, such as swabs [34].

For older age groups, the prevalence rate tends to approach zero, as observed in our pilot sample [8,34], primarily composed of healthy children with a mean age of 8 yrs. To our knowledge, this is the first study to apply a non-invasive oral sampling method in a general pediatric population without pre-selection based on maternal HPV status.

Upcoming phases of the HOPE project will investigate infection prevalence in a definitive sample of at least 500 subjects aged 0–14 years, with age groups stratified for homogeneity as follows: under 3 years, 4–7 years, 8–11 years, and over 11 years.

### 4.2. The Role of Social Determinants and Barriers to Awareness and Prevention of HPV Infection

The pilot questionnaire mostly involved the mothers of the recruited children with specific socio-economic and cultural conditions: married, homemakers, with low education and income levels, and at least two children to care for. The predominance of female parents or caregivers suggests that women are largely responsible for their children’s health (including oral health), with men taking on this role only in a minority of the cases. This trend may be rooted in cultural perceptions of gender roles within Sicilian families, which have traditionally followed a strongly patriarchal structure; this hypothesis requires further investigation through dedicated sociological or qualitative analysis.

Additionally, data on employment status (with over 68% of the parents or caregivers being housewives), education level (74% reporting education just above the primary level), religion (81% Catholic, both practicing and non-practicing), and income (86% indicating a low average socio-economic status) point to socio-economic and cultural barriers. These barriers may limit access to accurate and comprehensive knowledge about the causes of HPV transmission and effective preventive measures. Specifically, lower parental education levels and homemaker status were significantly associated with limited awareness of HPV and reduced vaccine uptake. These findings support the hypothesis that social disadvantage may hinder access to effective preventive strategies and underscore the need for targeted public health interventions.

Indeed, although a good percentage of the respondents (72.9%) reported knowledge of HPV, the modes of virus transmission were unclear, as is its association with cancers in the oro/ano-genital regions. Most of the sample did not know that there is no specific treatment for HPV, indicating limited awareness even regarding preventive strategies as the only effective means to combat the infection. Therefore, the knowledge of the existence of an HPV vaccine, as well as good awareness of the target population to be vaccinated, as reported by a sufficient portion of the respondents (54.3% and 50%), should be more thoroughly analyzed and pondered considering this datum.

It is not surprising that nearly all the parents who responded (94.3%) reported not being vaccinated and/or not remembering given their age. However, the attitude towards the future vaccination of their children is strongly influenced by the family doctor and/or pediatrician, who seems to play a key role in raising awareness and encouraging adherence to the HPV vaccination.

The physicians recommendations are the most influential facilitator for vaccination against HPV, with strong differences between the sexes, strongly correlated with previous information/awareness campaigns against HPV and the family context [37]. While physician and pediatrician recommendations clearly emerged as a key driver in parental willingness to vaccinate, other contextual factors may also influence vaccine-related decisions (i.e., educational attainment and socio-economic status).

Moreover, despite more than half of adolescents having sex by the age of 18, parents of younger adolescents generally tend to postpone vaccination to a later age; only when they perceive that their children are sexually active, or when targeted specialist visits (mainly gynecological) highlight the need for targeted prophylactic intervention through vaccination [38]. These findings align with previous evidence indicating that barriers to vaccine uptake are often multifactorial, involving informational, structural, and cultural dimensions.

To date, three vaccines are licensed and available worldwide: the bivalent Cervarix (GlaxoSmithKline, Brentford, UK) against HPV16 and HPV18, the quadrivalent Gardasil (Merck & Co. Inc, Rahway, NJ, USA) against HPV 6, 11, 16 and 18; and the nonavalent Gardasil9 (Merck & Co. Inc.) against HPV6, 11, 16, 18, 31, 33, 45, 52 and 58 [39]. In Italy, HPV vaccination has been part of the national immunization program since 2008, initially targeting girls aged 12. It is offered free of charge to prevent HPV-related cancers, including cervical cancer. Since 2017, the program expanded to include boys, aiming to further reduce HPV transmission and its impact [40,41,42]. In Sicily, HPV vaccination coverage has shown positive trends recently. For girls, coverage of the full vaccination cycle reached approximately 70% in 2022, though this remains below the 95% target for herd immunity. Coverage rates for boys are lower, with approximately 31% for those aged 12 and 13. Notably, regional disparities in vaccination rates persist across Italy, with southern regions like Sicily facing challenges related to socio-economic factors, healthcare access, and public awareness [26,43]. This alignment supports the hypothesis that the challenges identified in our cohort reflect broader regional patterns, driven by limited health education, socio-cultural barriers, and insufficient engagement with preventive care initiatives.

Additionally, although our pilot sample shows no statistically significant association between Catholic affiliation and HPV vaccine refusal, the influence of religion on vaccine uptake could be monitored.

A recent survey of over 400 Christian adults investigated how religious beliefs and teachings on sexual behavior may influence attitudes toward HPV vaccination for children. The findings suggest that some individuals perceive vaccination as unnecessary based on the belief that adherence to Catholic values and expected sexual conduct inherently provides protection against HPV infection [44].

Therefore, the predominance of Catholicism in Sicily and across Italy may influence parental beliefs regarding sexuality and disease prevention. Future phases of the HOPE project will explore this aspect more systematically in a larger and more diverse cohort [44].

The clustering of socio-economic disadvantage, low educational attainment, homemaker status, and limited income observed in our pilot sample may reflect broader structural barriers that hinder access to information, preventive services, and health-promoting behaviors. These patterns reflect the established frameworks of social vulnerability and health inequity, which emphasize the role of “systemic and structural determinants” in perpetuating disparities in health outcomes [45].

## 5. Limitations

The results should be interpreted considering the limitations inherent to the pilot study design, which was necessarily based on a small sample recruited in a single-center hospital setting. This context, along with the specific socio-cultural characteristics and healthcare access profiles of the local community, may not reflect the general pediatric population, potentially introducing selection bias and limiting the generalizability of the findings.

Second, the statistical analysis plan did not account for potential confounding factors or apply corrections for multiple comparisons. As a result, some observed associations may reflect spurious correlations rather than true causal relationships. This aspect is particularly important given the exploratory nature of the pilot phase and the limited sample size. In subsequent phases, multivariable modeling and appropriate statistical adjustments will be employed to strengthen analytical rigor and reduce the likelihood of type I errors.

Finally, the cross-sectional design does not allow for causal inferences or insights into temporal relationships among the variables examined. Longitudinal follow-up and more advanced modeling approaches will be necessary to investigate causal mechanisms and the persistence of oral HPV infections over time. Future phases of the HOPE project will address these concerns by involving a larger and more diverse sample, encompassing a wide range of geographic areas and socio-economic conditions

## 6. Conclusions

Human papillomavirus (HPV) is a known cause of several cancers. While vaccination has proven successful in significantly reducing the incidence of HPV-related cancers, including cervical and oropharyngeal cancers, the role of oral HPV infections in children remains a critical area of concern. Understanding how these infections may progress and their potential link to future cancers is essential. In the family context, parents and caregivers may not fully comprehend the risks of oral HPV infections or the potential for these infections to persist and lead to cancers later in life. This lack of knowledge may stem from a combination of cultural, educational, and socio-economic factors, including insufficient access to information, misinformation, and limited healthcare resources.

This pilot study represents the foundational phase of the HOPE (**H**uman papillomavirus **O**ral infection in **PE**diatric population) project, which aims to explore the feasibility of oral HPV surveillance in children while assessing parental awareness and preventive attitudes. The study highlights the relevance of adopting a multidisciplinary, prevention-oriented approach that integrates virological screening with socio-behavioral analysis.

The use of the LolliSponge™ device for non-invasive salivary collection demonstrated strong potential as an age-appropriate tool for pediatric populations, offering a practical alternative to conventional oral rinse methods. Its ease of use and high acceptability make it a valuable component for future large-scale studies and potential screening programs in early-life settings.

Although exploratory in nature, the findings provide important indications for the refinement of methodologies and the identification of knowledge gaps that may influence vaccine uptake and health behaviors. As the HOPE project progresses, the integration of innovative diagnostic strategies with targeted public health interventions may contribute to a broader understanding of oral HPV infection in children and inform the development of effective, context-sensitive prevention policies.

## Figures and Tables

**Table 1:** Data on the oral status of the children’s pilot sample.

Parameters	N	%
**Sample size**	70	100%
**Gender**		
Male	32	45.7%
Female	38	54.2%
**Age**		
0–4	9	12.8%
5–9	36	51.4%
10–14	25	35.7%
**Dentition**		
- Deciduous	18	25.7%
- Mixed	39	55.7%
- Permanent	13	18.6%
**Gingivitis**		
- Absent	24	34.3%
- Localized (BoP score ≥ 10%, ≤30%)	17	24.3%
- Generalized (BoP score > 30%)	29	41.4%
- Associated with orthodontic treatment	0	0%
**Caries**		
- Absent (deciduous dentition)	4	5.7%
- Absent (ICDAS 0/mixed or permanent dentitions)	6	8.6%
- ECC tipo I	8	11.4%
- ECC tipo II	6	8.6%
- ICDAS 0	1	1.4%
- ICDAS 1–2	10	14.3%
- ICDAS 3–4	20	28.6%
- ICDAS 5–6	15	21.4%

BoP: Bleeding on Probing; ECC: Early Childhood Caries; ICDAS: International Caries Detection and Assessment System.

**Table 2:** Knowledge of HPV infection, preventive strategies, and attitudes in the pilot population.

Question/Answer	N	%
**(For females only) Do you undergo gynecological examinations periodically?**		
- Yes, at least once a year	27	43.5%
- Yes, at least once every 3 years	5	8.1%
- Yes, rarely, only if necessary	30	48.4%
- No, only during childbirth	0	0%
- Not applicable, I am male	0	0%
**(For females only) Have you ever had a PAP test?**		
- No, but I recognise its importance for cervical cancer prevention	15	24.2%
- No, and I don’t think I need it	2	3.2%
- No, I am not aware of it	13	21.0%
- Yes, at least once every 3 years	32	51.6%
- Not applicable, I am male	0	0%
**Do you regularly visit the dentist?**		
- Never	4	5.7%
- Yes, at least once a year	22	31.4%
- Yes, at least once every 3 years	1	1.4%
- Yes, rarely, only if necessary	43	61.4%
**Have you ever heard of the Human Papillomavirus (HPV)?**		
- No	19	27.1%
- Yes	51	72.9%
**If YES, what are the transmission methods?** *		
- I am not aware	40	57.1%
- Between partners, through direct sexual contact	30	42.9%
- Indirectly sexually (through semen, blood, or infected saliva)	11	15.7%
- From mother to child across the placenta	5	7.1%
- From mother to child through childbirth	7	10.0%
- From mother to child through breastfeeding	2	2.9%
- Through sharing infected hygiene items or utensils	3	4.3%
- Through infected skin contact	0	0%
**Which sexual practices increase the risk of contracting HPV?** *		
- I am not aware	68	97.1%
- Deep kissing	4	5.7%
- Having many sexual partners	8	11.4%
- Unprotected sexual practices	21	30.0%
**Which non-sexual habits increase the risk of contracting HPV?** *		
- I am not aware	62	88.6%
- Sharing linens and clothing used by infected individuals	1	1.4%
- Blood transfusions/sharing infected needles	7	10.0%
- Voluptuary habits (tobacco, alcohol)	1	1.4%
- Childbirth	1	1.4%
**Do you know that HPV can cause cancer?**		
- No	42	60.0%
- Yes	28	40.0%
**If YES, which types of cancer?** *		
- Cervical cancer	27	38.6%
- Vulvar and vaginal cancer	10	14.3%
- Penile cancer	2	2.9%
- Oropharyngeal cancer	3	4.3%
- Anal cancer	1	1.4%
- Other cancers	4	5.7%
**HPV infection can be contracted from**		
- I am not aware	30	42.9%
- Women	3	4.3%
- Men	0	0%
- Both	37	52.9%
**Is there a medical therapy to cure HPV infection?**		
- I am not aware	46	65.7%
- No	9	12.9%
- Yes	15	21.4%
**Are you aware of the HPV vaccine?**		
- No	32	45.7%
- Yes	38	54.3%
**If YES, who should it be administered to?**		
- To everyone (M, F) between ages 11 and 12	19	50.0%
- To everyone (M, F), at any age	12	31.6%
- Only to females, between ages 11 and 12	7	18.4%
- Only to females, at any age	0	0%
**Are you vaccinated against HPV?**		
- No, I am unaware that a vaccine exists	3	4.3%
- No, I am not interested	3	4.3%
- No, I am not vaccinated	26	37.1%
- I cannot say if I am vaccinated	34	48.6%
- Yes	4	5.7%
- No, because it is not mandatory	0	0%
- No, I am afraid of the HPV vaccine	0	0%
- No, I am against the HPV vaccine	0	0%
- No, I am afraid of vaccinations in general	0	0%
- No, I am against vaccinations in general	0	0%
- No, after the COVID vaccine, I don’t want any other vaccinations	0	0%
**If YES, at what age did you complete the vaccination?**		
- By age 25	3	75.0%
- After age 25	1	25.0%
**Will you have your child vaccinated against HPV?**		
- No, I don’t think it’s necessary	3	4.3%
- No, I am against vaccinations in general for them	3	4.3%
- No, after the COVID vaccine, I don’t want my children to have any other vaccinations	1	1.4%
- I don’t have enough information to decide right now (I will decide when advised by doctors)	32	45.7%
- Yes, I will vaccinate them when they request it	1	1.4%
- Yes, I will vaccinate them between ages 11 and 12 (as outlined in the optional vaccination plan)	30	42.9%
- No, because it is not mandatory	0	0%
- No, I am afraid of the HPV vaccine for them	0	0%
- No, I am against the HPV vaccine for them	0	0%
- No, I am afraid of vaccinations in general for them	0	0%

* note: multiple answers were considered.

**Table 3:** Statistically significant association between socio-demographic factors and oral health status in children (gingivitis).

Child’s Gingivitis	Absent (*n* = 24)	Present (*n* = 46)	OR	95%CI	*p*-Value
**Child’s Dentition**					
Deciduous	11	7	-	-	-
Mixed	10	29	4.38	1.35–15.40	**0.013**
Permanent	3	10	4.82	1.03–29.44	**0.046**
**Occupation**					
Homemaker	10	38	-	-	-
Unemployed	4	2	0.14	0.01–0.88	**0.036**
Employee	6	4	0.18	0.03–0.78	**0.022**
Self-employed	4	2	0.14	0.01–0.88	0.036

The bold formatting is used to highlight values with statistical significance.

**Table 4:** Statistically significant association between socio-demographic factors and parental attitudes toward oral healthcare.

Dental Visits	Never/Rarely (*n* = 47)	Yes (*n* = 23)	OR	95%CI	*p*-Value
**Child’s Dentition**					
Deciduous	9	9	-	-	-
Mixed	28	11	0.4	0.12–1.29	0.126
Permanent	10	3	0.32	0.05–1.50	0.153
**Parent’s Age**					
18–33	13	9	-	-	-
34–41	18	12	0.96	0.31–3.04	0.947
42+	16	2	0.19	0.02–0.95	**0.043**
**Religion**					
Other	9	4	-	-	-
Catholic	38	19	1.1	0.30–4.67	0.882

The bold formatting is used to highlight values with statistical significance.

**Table 5:** Statistically significant associations between socio-demographic factors and knowledge of vaccine’s existence.

Are you Aware of the HPV Vaccine?	No (*n* = 32)	Yes (*n* = 38)	OR	95%CI	*p*-Value
**Occupation**					
Homemaker	20	28	-	-	-
Unemployed	5	1	0.16	0.01–1.16	0.073
Employee	2	8	2.68	0.57–21.16	0.223
Self-employed	5	1	0.16	0.01–1.16	0.073

**Table 6:** Statistically significant associations between socio-demographic factors and HPV vaccination status of relatives.

Are You Vaccinated (Mother)?	No (*n* = 32)	Not Sure/Yes (*n* = 38)	OR	95%CI	*p*-Value
**Education Level**					
High	14	4	-	-	-
Low	18	34	6.28	1.90–25.64	**0.001**
**Parental Status**					
Married	24	27	-	-	-
Cohabiting	3	6	1.72	0.39–9.45	0.477
Separated/Divorced	3	1	0.32	0.01–3.02	0.342
Single	2	3	1.29	0.18–11.95	0.793
Widowed	0	1	N.S.	N.S.	N.S.
**Number of Children**					
≥3	16	16	-	-	-
1–2	16	22	1.36	0.53–3.59	0.521

## Data Availability

The original contributions presented in this study are included in the article. Further inquiries can be directed to the corresponding author.
